# Glycerol Monolaurate Supplementation in Extruded Diets Enhances Immune Function and Antioxidant Capacity and Alters the Gut Microbiota and Fecal Metabolome in Cats

**DOI:** 10.3390/antiox15091169

**Published:** 2026-09-15

**Authors:** Haotian Jiang, Bo Liu, Jialin Zhang, Xin Ge, Zhonghui Jin, Shuaijuan Han, Shudong Liu

**Affiliations:** 1 College of Animal Science and Technology, Hebei Agricultural University, Baoding 071001, China; 2 Beijing Zhongnong Lifeng Biotechnology Co., Ltd., Beijing 102208, China

**Keywords:** cats, extruded diet, glycerol monolaurate, microbiome, metabolomics

## Abstract

This study evaluated the effects of dietary glycerol monolaurate (GML) supplementation on feline growth performance and intestinal health. Twenty-four adult British Shorthair cats (3.03 ± 0.07 kg) were initially enrolled. Following a 56-day pre-feeding period, 18 eligible cats were allocated to two dietary groups, of which 12 cats were prespecified for final sample collection and statistical analysis (*n* = 6): a control group (CON, basal diet) and an experimental group (EXP, basal diet + 2000 mg/kg GML product) for a 28-day trial. Dietary GML significantly increased the average daily feed intake. GML supplementation significantly increased the changes in fecal score and fecal pH, and a significant GML × day interaction was observed for the change in fecal pH. GML improved the apparent digestibility of dry matter, gross energy, crude fat, and crude protein. Furthermore, serum albumin increased within the normal range. Cats in the EXP group showed elevated antioxidant enzyme activities, alongside lower malondialdehyde concentrations. GML also elevated serum immunoglobulin A and reduced pro-inflammatory cytokines. Fecal analyses showed reduced acetic acid and total volatile fatty acids, but elevated propionic acid. Microbiome and metabolomic profiling revealed that GML decreased the relative abundance of *Streptococcus* and metabolites such as Linoleic Acid and 12,13-Epome, while increasing the relative abundances of *Collinsella*, *Peptoclostridium*, *Clostridium*, and (-)-Jasmonic Acid. These findings provide a theoretical foundation for applying GML in pet foods to optimize companion animal extruded diets.

## 1. Introduction

With rising living standards and evolving demographic and household structures, the companion animal population is steadily increasing, making pet ownership an increasingly common lifestyle choice. A pet ownership survey covering 20 countries worldwide, published by Mars Incorporated in 2024, revealed that the global pet population is estimated to exceed one billion, with cat ownership being more common than dog ownership [1]. This situation may be related to the fact that domestic cats are better adapted to urban living environments, as most of them do not require outdoor activities. Driven by growing awareness of pet welfare, owners typically provide their pets with a nutritionally diverse diet. Cats are obligate carnivores, and their dietary requirement for protein is higher than that of dogs [2]. However, because many pet owners are busy with work and unable to feed fresh meat, the majority choose to feed their cats commercial diets. The mainstream commercial diets on the market are divided into extruded diets and baked diets, among which extruded diets are widely favored by consumers because of their relatively low production costs and convenient processing and storage characteristics. However, whether the long-term feeding of extruded diets is entirely healthy for cats remains a subject of concern. In fact, extrusion technology has been applied in the food processing industry since 1930 and is already a well-established manufacturing technology [3]. Nevertheless, despite the carnivorous characteristics of cats, a certain proportion of starch (primarily derived from cereals) is usually added to commercial extruded cat diets, which is a necessity for the extrusion and expansion process of manufacturing cat food [4]. However, because cats lack salivary amylase and exhibit relatively low activities of pancreatic amylase and intestinal disaccharidases [5,6], their capacity to utilize carbohydrates such as starch is limited. Excessive carbohydrates can also exert adverse effects on cats; for example, high carbohydrate intake may reduce protein digestibility and increase the amount of undigested carbohydrates reaching the colon, thereby enhancing colonic microbial fermentation and organic acid production [7]. This potential risk associated with the long-term feeding of extruded diets poses a challenge to the pet food system. Accordingly, optimizing starch inclusion levels or developing novel feed additives to mitigate the adverse effects of dietary starch and other carbohydrates in cats has become an important research priority in companion animal nutrition.

Glycerol monolaurate (GML) is a monoglyceride ester derived from lauric acid and glycerol, possessing multiple biological activities and physiological functions. Its safety has been recognized by the U.S. Food and Drug Administration, and it can be used as a food additive within a dosage range of 10 to 2000 mg/kg [8]. In recent years, studies on various animals have found that GML can not only serve as a readily available energy source for the animal but also exert multiple physiological effects, including modulation of the intestinal microbiota and promotion of intestinal development. In addition, GML has been reported to possess antibacterial and anti-inflammatory properties and to enhance both immune function and antioxidant capacity in animals [9,10,11]. However, studies on GML in companion animals remain limited. Therefore, the present study was conducted to investigate whether dietary GML supplementation can alleviate the adverse effects induced by long-term feeding of extruded diets in domestic cats. This study aimed to clarify the effects of GML on the intestinal health, antioxidant capacity, and immune function of pet cats, providing a reference for its scientific application in pet diets.

## 2. Materials and Methods

### 2.1. Ethics Approval and Consent to Participate

This study was conducted in 2026 at the Livestock Teaching and Research Base of Hebei Agricultural University, Baoding, China. All experimental procedures were approved by the Hebei Agricultural University Institutional Animal Care and Use Committee (approval number: 2026152).

### 2.2. Materials

The GML product used in the current experiment was supplied by Adisseo Life Science (Shanghai) Co., Ltd., Shanghai, China (batch No. 09062025). The product was formulated with precipitated and dried silica as the carrier, and its main components included GML, glycerol, and water. Per the manufacturer’s guaranteed analysis, the product contained 54% GML, 7% glycerol, 9% moisture, and 30% crude ash.

### 2.3. Preparation of Experimental Diets

The ingredient composition and nutrient levels of the experimental diets are shown in Table 1. The formulated diets met the nutritional requirements of healthy adult cats according to the Association of American Feed Control Officials guidelines [12]. The basal diet was first preconditioned at approximately 67 °C and subsequently processed by extrusion cooking. During extrusion, the screw speed was maintained at approximately 350 rpm, while the die temperature and pressure were approximately 145 °C and 350 psi, respectively. After extrusion, the kibbles were dried at 115.5 °C until the moisture content was below 10% and then cooled before coating. The additive (GML) was mixed with chicken fat, feline flavor digest, and chicken liver powder at a preset ratio to formulate a uniform coating slurry. This slurry was uniformly applied onto the kibble surface by atomization using specialized spraying equipment, followed by secondary low-temperature drying and curing. This procedure protected the GML product from thermal degradation during processing while ensuring uniform adhesion and stable retention on the kibble surface.

### 2.4. Experimental Design, Animals, and Management

A total of 24 healthy adult British Shorthair cats (body weight 3.03 ± 0.07 kg; half male and half female) were initially enrolled in this study. Prior to the formal experiment, a 56-day pre-feeding period was conducted, during which all cats were fed the basal extruded diet. Cats were considered eligible for the subsequent experiment if they maintained stable feed intake, showed no excessive body weight gain, had fecal scores of ≥3, and remained clinically healthy throughout the pre-feeding period. Following the pre-feeding period, 18 cats that met these eligibility criteria were included in the subsequent 28-day experiment. These cats were stratified according to body weight and sex and then randomly allocated to the control group (CON) and experimental group (EXP). Before the beginning of the formal experiment, six cats in each group were randomly designated for final sample collection and statistical analysis, while three additional cats in each group were maintained as reserve animals in case any of the designated animals had to be withdrawn because of illness or other animal welfare concerns. The three reserve cats in each group were subjected to the same feeding, housing, health monitoring, and routine management procedures as the six designated cats throughout the experimental period. There was no significant difference in initial body weight (IBW) between the two groups (*p* > 0.05). All personnel involved in the experimental procedures and outcome assessments were blinded to the treatment allocation throughout the study. The CON group received the basal extruded diet, whereas the EXP group received the basal diet supplemented with 2000 mg/kg of the GML product. Throughout the trial, fresh feed was provided daily at 08:00, and all cats had ad libitum access to drinking water. During the experimental period, the daily feed intake (g/d) and clinical health status of each cat were monitored and recorded. The amount of feed offered was calculated according to the daily energy requirement formula for adult cats at maintenance provided by the National Research Council: 100 × (BW)^0.67^ = kcal/day [13]. Regarding feed intake, all cats consumed the recommended rations required for body weight maintenance. Prior to the initiation of the experiment, all cats were vaccinated and dewormed, and none had received any medications (e.g., antibiotics) that could potentially alter the intestinal microbiota. The cats were individually housed in cages (2 × 0.7 × 0.6 m^3^). The ambient room temperature was maintained at 23 to 25 °C, with a relative humidity of 40% to 60%. To ensure animal welfare, each cat was released from its cage for 1 h daily for voluntary activity and exercise and was provided with various toys and regular human interactions. Cages and litter boxes were regularly cleaned and disinfected.

### 2.5. Determination of Growth Performance

The fasting body weight of each cat was measured at 08:00 on days 1, 7, 14, 21, and 28 of the trial (feed and water were withheld from 20:00 on the previous day). Throughout the experimental period, the amounts of newly provided feed and the remaining feed from the previous day were recorded daily at 08:00. Subsequently, the average daily feed intake (ADFI) and average daily gain (ADG) were calculated. Additionally, the changes in body weight on days 7, 14, 21, and 28 relative to day 1 were determined.

### 2.6. Body and Fecal Scores

The body condition score (BCS), muscle condition score (MCS), body fat index (BFI), and fecal score (FS) of each cat were evaluated at 08:00 on days 1, 7, 14, 21, and 28 of the trial. The BCS and MCS were assessed according to the criteria established by Baldwin et al. Specifically, a 9-point scoring system was used for the BCS (scores 1–3 indicate an under-ideal body condition, 4–5 indicate an ideal body condition, and 6–9 indicate an over-ideal body condition), while a 4-point scoring system was used for the MCS (score 1 indicates marked muscle wasting, 2 indicates moderate muscle wasting, 3 indicates mild muscle wasting, and 4 indicates no muscle wasting, normal muscle mass). The BFI was scored based on the criteria established by Witzel et al. using a BFI risk chart, where a score of 20 (16–25% body fat) is classified as low risk, 30 (26–35% body fat) as moderate risk, 40 (36–45% body fat) as high risk, 50 (46–55% body fat) as serious risk, 60 (56–65% body fat) as severe risk, and 70 (66–75% body fat) as extreme risk. The FS was evaluated according to the Royal Canin fecal scoring system, ranging from 1 to 5, with an intermediate score of 4.5. Scores of 1, 2, 3, 4, 4.5, and 5 represented watery, very soft, soft but formed, well-formed and slightly moist, well-formed and dry, and hard and dry feces, respectively. When fecal consistency varied within a sample, the lower score was recorded. Fecal pH was measured at three randomly selected locations within each sample using a Testo-205 pH meter (Testo AG, Titisee-Neustadt, Germany). After all scores were recorded, the changes in these scores on days 7, 14, 21, and 28 relative to day 1 were calculated.

### 2.7. Nutrient Digestibility

On days 1, 7, 14, 21, and 28 of the trial, 100 g of diet samples were collected from each group. At the end of the trial, the diet samples from each group were mixed and stored in a desiccator for subsequent analysis. Starting from day 25 of the trial, fresh feces from each cat were collected continuously for 3 days. The feces were retrieved before the cats performed the litter-burying behavior. After removing hair, cat litter, and dust from the feces, a 10% hydrochloric acid solution was added at 5% of the fresh weight for nitrogen preservation. The treated feces from each cat were then placed in an individual sealable bag and stored at −20 °C. At the end of the trial, the 3-day fecal samples from each cat were uniformly mixed and placed in an oven at 65 °C for 48 h. After drying, the samples were ground and passed through a 40-mesh sieve for the determination of nutrient composition.

The endogenous indicator method was used to evaluate the apparent digestibility of nutrients in each group of cats. Analyses of feed and fecal samples followed the requirements of AOAC (2019) [14]. Dry matter (DM) was determined according to method 930.15 of AOAC (2019) [14]. Crude protein (CP) content was determined using a Kjeldahl nitrogen analyzer (KT8400; FOSS Analytical AB, Höganäs, Sweden) according to method 990.03 in AOAC (2019) [14]. Ether extract (EE) was measured using a fat analyzer (FoodALYTRD40, OMNILAB, Bremen, Germany) according to method 945.16 of AOAC (2019) [14]. Calcium (Ca) and phosphorus (P) concentrations were determined using an atomic absorption spectrophotometer (ZA4000; Hitachi High-Technologies, Tokyo, Japan) according to method 985.01 of AOAC (2019) [14]. Gross energy (GE) content was analyzed using a bomb calorimeter (Model 6300; Parr Instruments, Moline, IL, USA). The amino acid composition of the diet samples was determined by ultra-performance liquid chromatography (LA8080; Hitachi High-Technologies, Tokyo, Japan) according to method 982.30 of AOAC (2019) [14].

### 2.8. Fecal Sample Collection and Analysis

On day 28 of the trial, fresh feces from the cats were collected. After the feces were circumferentially excised using a pre-treated sterile scalpel, the inner core of the feces was placed into 5-mL sterile fecal collection tubes and frozen at −80 °C for subsequent use.

### 2.9. Volatile Fatty Acids (VFAs)

A 0.2-g fecal sample was taken from −80 °C storage and placed into a 1.5-mL centrifuge tube. Subsequently, 1 mL of pre-cooled ultrapure water was added, and the mixture was vortexed. The sample was then centrifuged at 5000× *g* for 10 min at 4 °C using a refrigerated centrifuge (L535R; Xiangyi Centrifuge Instrument, Changsha, China). The resulting supernatant was vortex-mixed with 1 mL of 25% metaphosphoric acid for 30 s, incubated in an ice bath for 40 min, and centrifuged again under the same conditions. Finally, the supernatant was collected, and the concentrations of acetic, propionic, isobutyric, butyric, isovaleric, and valeric acids were determined using a gas chromatograph (7890A; Agilent Technologies, Santa Clara, CA, USA). The total volatile fatty acid (TVFA) concentration was then calculated.

### 2.10. Assessment of Microbial Diversity

The fecal samples were retrieved from −80 °C storage, and microbial DNA was extracted using the E.Z.N.A.^®^ Soil DNA Kit (Omega Bio-tek, Norcross, GA, USA). The V3–V4 hypervariable region of the 16S rRNA gene was amplified by PCR using the primers 338F (5′-ACTCCTACGGGAGGCAGCAG-3′) and 806R (5′-GGACTACHVGGGTWTCTAAT-3′). PCR was carried out in a total volume of 20 μL containing 4 μL of 5× FastPfu buffer, 2 μL of 2.5 mM dNTPs, 0.8 μL of each primer (5 μM), 0.4 μL of FastPfu polymerase, approximately 10 ng of template DNA, and ddH_2_O. Thermal cycling consisted of an initial denaturation at 95 °C for 3 min, followed by 27 cycles at 95 °C for 30 s, 55 °C for 30 s, and 72 °C for 45 s, and a final extension at 72 °C for 10 min. Sequencing was performed by Shanghai Majorbio Bio-pharm Technology Co., Ltd. (Shanghai, China) on an Illumina MiSeq platform (Illumina, Inc., San Diego, CA, USA) using a paired-end 300-bp sequencing strategy. The specific bioinformatics analyses were conducted as follows: Alpha diversity indices were calculated using the mothur software (version 1.48.5), and inter-group differences in alpha diversity were analyzed using the Wilcoxon rank-sum test. Principal coordinate analysis (PCoA) based on Bray–Curtis distances was used to evaluate the similarity of microbial community structures among samples, and non-parametric tests were applied to determine the significance of these structural differences between groups. Venn diagrams were generated using the R package VennDiagram (version 1.8.2) to visualize shared and unique amplicon sequence variants (ASVs) among samples or groups. The relative abundances of the microbial communities from the phylum to species levels were calculated via raw sequence analysis, DADA2, and VSEARCH analyses using QIIME 2. Finally, linear discriminant analysis effect size (LEfSe) (LDA > 2, *p* < 0.05) was used to identify bacterial taxa with significantly different abundances from the phylum to genus levels among different groups.

### 2.11. Untargeted Metabolomics

Fecal untargeted metabolomics was conducted to extract and analyze metabolites in the fecal samples using an ultra-high-performance liquid chromatography–tandem mass spectrometry (UHPLC-Q Exactive HF-X) system (Thermo Fisher Scientific, Waltham, MA, USA) by Shanghai Majorbio Bio-pharm Technology Co., Ltd. (Shanghai, China). Chromatographic separation was performed using an HSS T3 column (2.1 × 100 mm, 1.8 μm; Waters Co., Milford, MA, USA) at a flow rate of 0.4 mL/min, a column temperature of 40 °C, and an injection volume of 3 μL. Partial least-squares discriminant analysis (PLS-DA) was performed using the ropls package (version 1.6.2) in R software (version 4.6.1) to explore the differences in metabolite profiles between groups. Based on a variable importance in projection (VIP) > 1 and *p* < 0.05, relative abundance analysis of differentially enriched metabolites, clustering analysis of differential metabolites, and Kyoto Encyclopedia of Genes and Genomes (KEGG) metabolic pathway analysis were conducted. The enriched pathways were visualized using the KEGG Mapper tool to generate differential metabolite and pathway diagrams.

### 2.12. Serum Sample Collection and Analysis

One day after fecal collection, forelimb venous blood was collected from all cats following a 12-h fast. A 2-mL volume of venous blood was collected into a K_2_-EDTA anticoagulant vacuum tube, and the hematological indices in the blood samples were determined using an automated hematology analyzer (URIT-5160VET; URIT, Guilin, China). The measured parameters included white blood cell count (WBC), lymphocyte count (Lymph#), lymphocyte percentage (Lymph%), monocyte count (Mon#), monocyte percentage (Mon%), granulocyte count (Gran#), granulocyte percentage (Gran%), eosinophil percentage (Eos%), mean corpuscular volume (MCV), mean corpuscular hemoglobin (MCH), mean corpuscular hemoglobin concentration (MCHC), red blood cell distribution width (RDW), red blood cell count (RBC), hemoglobin concentration (HGB), hematocrit (HCT), platelet count (PLT), mean platelet volume (MPV), platelet distribution width (PDW), and plateletcrit (PCT). Additionally, 5 mL of venous blood was collected into a procoagulant vacuum tube. After standing for 30 min, the blood sample was centrifuged at 3500× *g* for 15 min. The resulting serum was aliquoted into 1.5-mL centrifuge tubes and stored at −80 °C for subsequent use. The levels of blood urea nitrogen (BUN), total protein (TP), albumin (ALB), globulin (GLB), total cholesterol (TC), triglycerides (TG), high-density lipoprotein (HDL), alkaline phosphatase (ALP), aspartate aminotransferase (AST), alanine aminotransferase (ALT), total bilirubin (TBIL), and gamma-glutamyl transferase (γ-GT) were determined using a fully automated biochemical analyzer (BS-420; Mindray, Shenzhen, China). Total antioxidant capacity (T-AOC), superoxide dismutase (SOD), glutathione peroxidase (GSH-Px), catalase (CAT), malondialdehyde (MDA), immunoglobulin A (IgA), immunoglobulin G (IgG), immunoglobulin M (IgM), tumor necrosis factor-α (TNF-α), interleukin-6 (IL-6), and interleukin-8 (IL-8) were measured using enzyme-linked immunosorbent assay (ELISA) kits (Beijing Sino-UK Institute of Biological Technology, Beijing, China) according to the manufacturer’s instructions.

### 2.13. Statistical Analysis

Data other than microbiota and metabolomics data were analyzed using SPSS 26.0 (IBM Corp., Armonk, NY, USA). Independent-samples *t*-tests or two-way repeated-measures analysis of variance were used as appropriate. In the two-way repeated-measures analysis of variance, GML was the between-subject factor and Day was the within-subject factor. Fisher’s protected least significant difference test was used for post hoc comparisons. Significant differences in the microbiota were analyzed by non-parametric tests using GraphPad Prism software (version 10.6.1; GraphPad Software, San Diego, CA, USA). For metabolomics data, the significance of differential metabolites was evaluated by calculating *p*-values, VIP values, and fold changes (FC). Spearman correlation analysis was performed, and the data were imported into the Genes Cloud platform (https://www.genescloud.cn/) for analysis. Data are presented as the mean ± standard error of the mean. A value of *p* < 0.05 was considered statistically significant, 0.05 ≤ *p* < 0.10 was considered indicative of a statistical trend, and *p* ≥ 0.10 was considered non-significant. Metabolites with *p* < 0.05 and VIP > 1 were considered statistically significant.

## 3. Results

### 3.1. Effects of the GML Product on the Growth Performance of British Shorthair Cats

As shown in Table 2, the addition of the GML product to the diet significantly increased the ADFI of cats (*p* < 0.05). Although the ADG was increased by 43.99%, no statistically significant difference was observed (*p* > 0.05).

### 3.2. Effects of the GML Product on the Body and Fecal Parameters of British Shorthair Cats

As shown in Table 3, there were no statistically significant differences in the body weight, BCS, MCS, BFI, and FS between the two groups on day 1 of the trial (*p* > 0.05). On day 28 of the trial, the FBW of cats in the experimental group showed a trend toward an increase compared with the control group (*p* = 0.065). As shown in Table 4, a significant dietary GML supplementation × Day interaction was observed for ΔpH (*p* < 0.05). In cats fed the GML-supplemented diet [GML(+)], ΔpH increased progressively from d7 to d28, and all time points differed from one another (*p* < 0.05). Compared with the control group [GML(−)], the GML-supplemented group showed higher ΔpH values on d14, d21 and d28 (*p* < 0.05), whereas no difference was observed on d7. Across time points, ΔFS was higher in the GML-supplemented group than in the control group (*p* = 0.0499). ΔBFI differed among days (*p* = 0.048), with higher values on d21 and d28 than on d7 (*p* < 0.05). ΔBW also differed among days (*p* < 0.05), with values on d14, d21 and d28 being higher than that on d7 (*p* < 0.05); additionally, the value on d28 was higher than that on d14 (*p* < 0.05).

### 3.3. Effects of the GML Product on Apparent Nutrient Digestibility of British Shorthair Cats

As shown in Table 5, the addition of the GML product to the diet significantly increased the apparent digestibility of dry matter, energy, fat, and protein in cats (*p* < 0.05).

### 3.4. Effects of the GML Product on the Hematological Indices of British Shorthair Cats

As shown in Table 6, the addition of the GML product to the diet led to a trend toward a decrease in the WBC in the blood of cats (*p* = 0.091), whereas no significant differences were observed in the remaining hematological indices (*p* > 0.05). Furthermore, all indices were within the normal range.

### 3.5. Effects of the GML Product on the Serum Biochemical Indices of British Shorthair Cats

As shown in Table 7, the addition of the GML product to the diet significantly increased the serum ALB concentration of cats in the experimental group, while the values remained within the normal range (*p* < 0.05), whereas the concentrations of TC (*p* = 0.061) and TG (*p* = 0.068) showed a trend toward a decrease. No significant differences were observed in the remaining indices (*p* > 0.05), and all indices were within the normal range.

### 3.6. Effects of the GML Product on the Serum Antioxidant Function of British Shorthair Cats

As shown in Table 8, the addition of the GML product to the diet significantly increased serum T-AOC and the activities of SOD, CAT, and GSH-Px in the serum of cats in the experimental group (*p* < 0.05) and significantly decreased the serum MDA content (*p* < 0.05).

### 3.7. Effects of the GML Product on the Serum Immune Function of British Shorthair Cats

As shown in Table 9, the addition of the GML product to the diet significantly increased the IgA concentration (*p* < 0.05) and significantly decreased the serum TNF-α, IL-6, and IL-8 concentrations (*p* < 0.05) of cats in the experimental group.

### 3.8. Effects of the GML Product on the Fecal Volatile Fatty Acids of British Shorthair Cats

As shown in Table 10, the addition of the GML product to the diet significantly decreased the fecal acetic acid and TVFA content (*p* < 0.05) and significantly increased the fecal propionic acid content (*p* < 0.05) of cats in the experimental group.

### 3.9. Effects of the GML Product on the Fecal Microbiota of British Shorthair Cats

**Figure 1:**
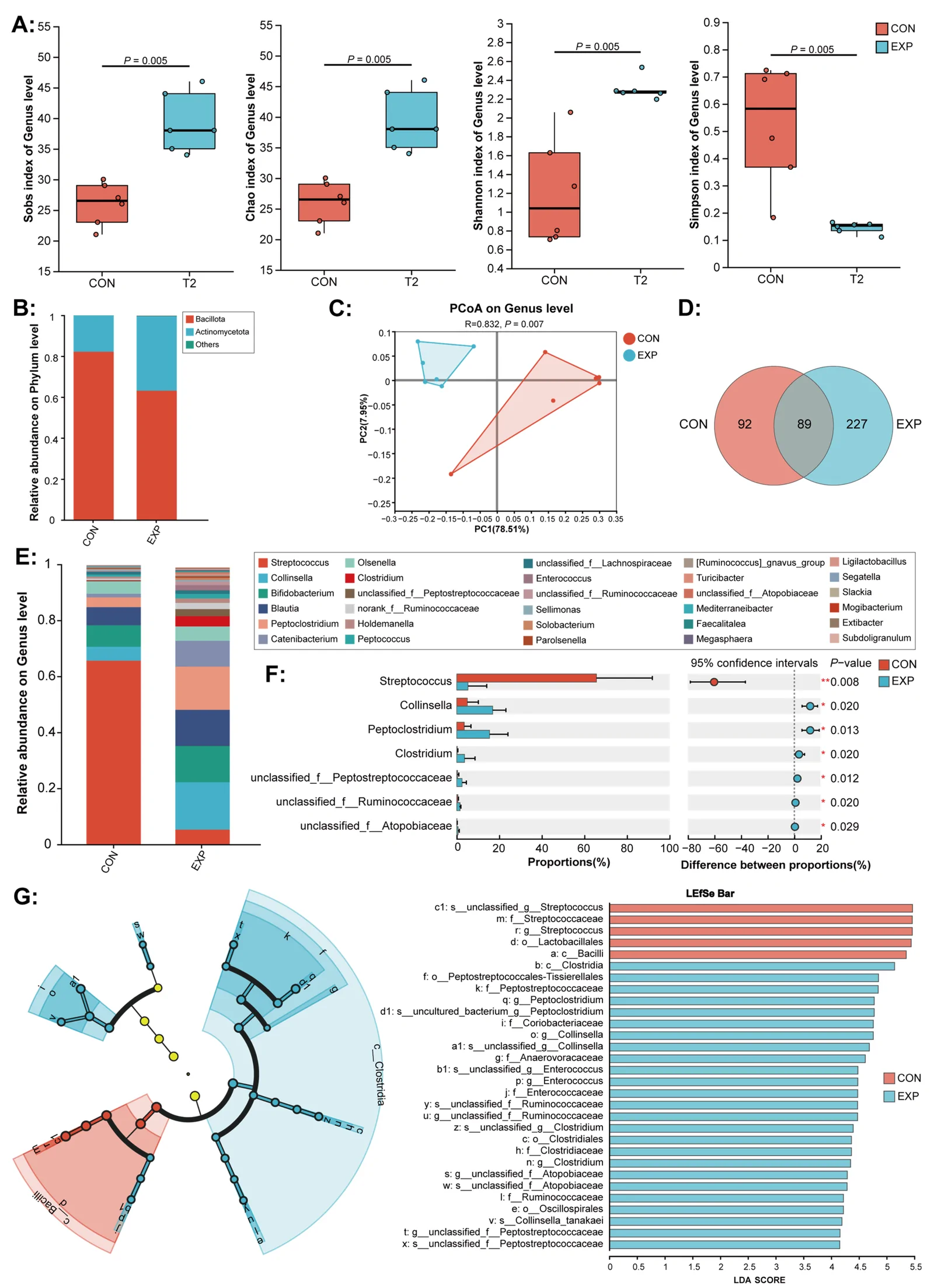
Effects of the GML product on the fecal microbiota of British Shorthair cats. (**A**) Alpha diversity; (**B**) taxonomic composition at the phylum level; (**C**) principal coordinate analysis; (**D**) Venn diagram of amplicon sequence variants; (**E**) taxonomic composition at the genus level; (**F**) comparative analysis between the two groups; (**G**) LEfSe analysis. CON: control group; EXP: experimental group. * indicates a significant difference at *p* < 0.05. ** indicates a significant difference at *p* < 0.01.

As shown in Figure 1A, the addition of the GML product to the diet significantly increased the Sobs, Chao, and Shannon indices of the fecal microbiota in cats (*p* < 0.05) and significantly decreased the Simpson index (*p* < 0.05). As indicated by the results of the principal coordinate analysis shown in Figure 1C, principal coordinate 1 and principal coordinate 2 accounted for 78.51% and approximately 7.95% of the variation in the fecal microbial community, respectively. Together, they accounted for 86.46% of the total variation. Furthermore, PCoA showed a distinct separation between the CON and EXP groups, and ANOSIM confirmed a significant difference in microbial community structure between the two groups (R = 0.832, *p* = 0.007)., indicating that supplementation with the GML product had a significant impact on the fecal microbiota of cats. As shown in Figure 1D, there were 89 ASVs shared between the CON and EXP groups, whereas 92 and 227 ASVs were unique to the CON and EXP groups, respectively. As illustrated in Figure 1B, the fecal microbiota of cats at the phylum level was primarily composed of Bacillota and Actinomycetota, with the sum of these two phyla accounting for 99.84% and 99.70% in the CON and EXP groups, respectively. As shown in Figure 1E,F, the abundance of *Streptococcus* in the EXP group was significantly lower than that in the CON group (*p* < 0.05), whereas the abundances of *Collinsella*, *Peptoclostridium*, *Clostridium*, *unclassified_f__Peptostreptococcaceae*, *unclassified_f__Ruminococcaceae*, and *unclassified_f__Atopobiaceae* were significantly higher in the EXP group than in the control group (*p* < 0.05). Further LEfSe analysis using a “one-against-all” approach to evaluate the differences between the two groups (LDA > 2.0) revealed that, as shown in Figure 1G, the abundances of taxa such as *g__Streptococcus*, *o__Lactobacillales*, and *c__Bacilli* were significantly decreased in the experimental group compared with the control group (*p* < 0.05), whereas the abundances of taxa such as *f__Peptostreptococcaceae*, *g__Clostridium*, and *s__Collinsella tanakaei* were significantly increased (*p* < 0.05).

### 3.10. Effects of the GML Product on the Fecal Metabolites of British Shorthair Cats

**Figure 2:**
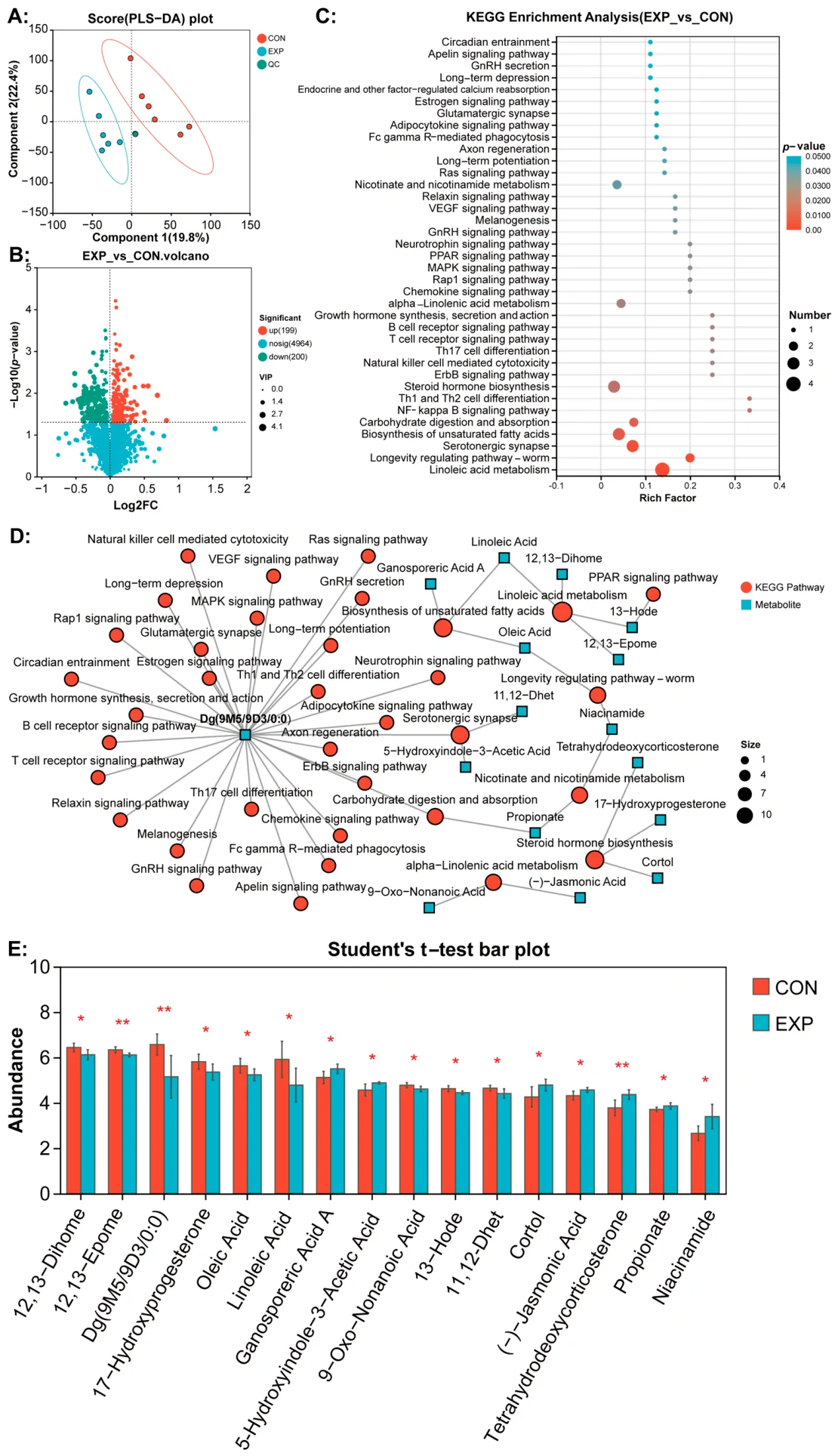
Effects of the GML product on the fecal metabolites of British Shorthair cats. (**A**) PLS-DA score plot; (**B**) volcano plot; (**C**) KEGG enrichment analysis; (**D**) KEGG enrichment network; (**E**) Student’s *t*-test bar plot of the significantly enriched metabolites in the network. CON: control group; EXP: experimental group. * indicates a significant difference at *p* < 0.05. ** indicates a significant difference at *p* < 0.01.

As shown in the PLS-DA score plot (Figure 2A), the sample points of the CON and EXP groups were clearly separated with no overlap in their 95% confidence intervals, indicating that the addition of the GML product to the diet significantly altered the fecal metabolic profile of British Shorthair cats. The volcano plot (Figure 2B) revealed that, compared with the CON group, 199 differentially abundant metabolites (DAMs) were significantly up-regulated, and 200 DAMs were significantly down-regulated in the EXP group (*p* < 0.05, VIP > 1). The KEGG pathway enrichment analysis (Figure 2C) demonstrated that the DAMs were mainly enriched in pathways related to linoleic acid metabolism, steroid hormone biosynthesis, biosynthesis of unsaturated fatty acids, and carbohydrate digestion and absorption, as well as several pathways associated with immunity and inflammation. The KEGG enrichment network (Figure 2D) further illustrated the associations between key DAMs and these pathways; notably, Dg(9M5/9D3/0:0) was linked to 28 significantly altered metabolic pathways. Additionally, various DAMs such as Propionate, Linoleic Acid, and Oleic Acid were also associated with multiple metabolic pathways. The Student’s *t*-test bar chart (Figure 2E) presents the relative abundances of the DAMs enriched in the significantly changed metabolic pathways. For example, compared with the CON group, the level of Dg(9M5/9D3/0:0), a DAM enriched in various immune and inflammatory pathways, was significantly decreased in the EXP group. Similarly, the levels of Linoleic Acid, 12,13-Epome, 13-Hode, and 12,13-Dihome, which were enriched in the Linoleic acid metabolism pathway, were significantly decreased. Conversely, the level of Propionate, which was enriched in the Carbohydrate digestion and absorption and the Nicotinate and nicotinamide metabolism pathways, was significantly increased.

**Figure 3:**
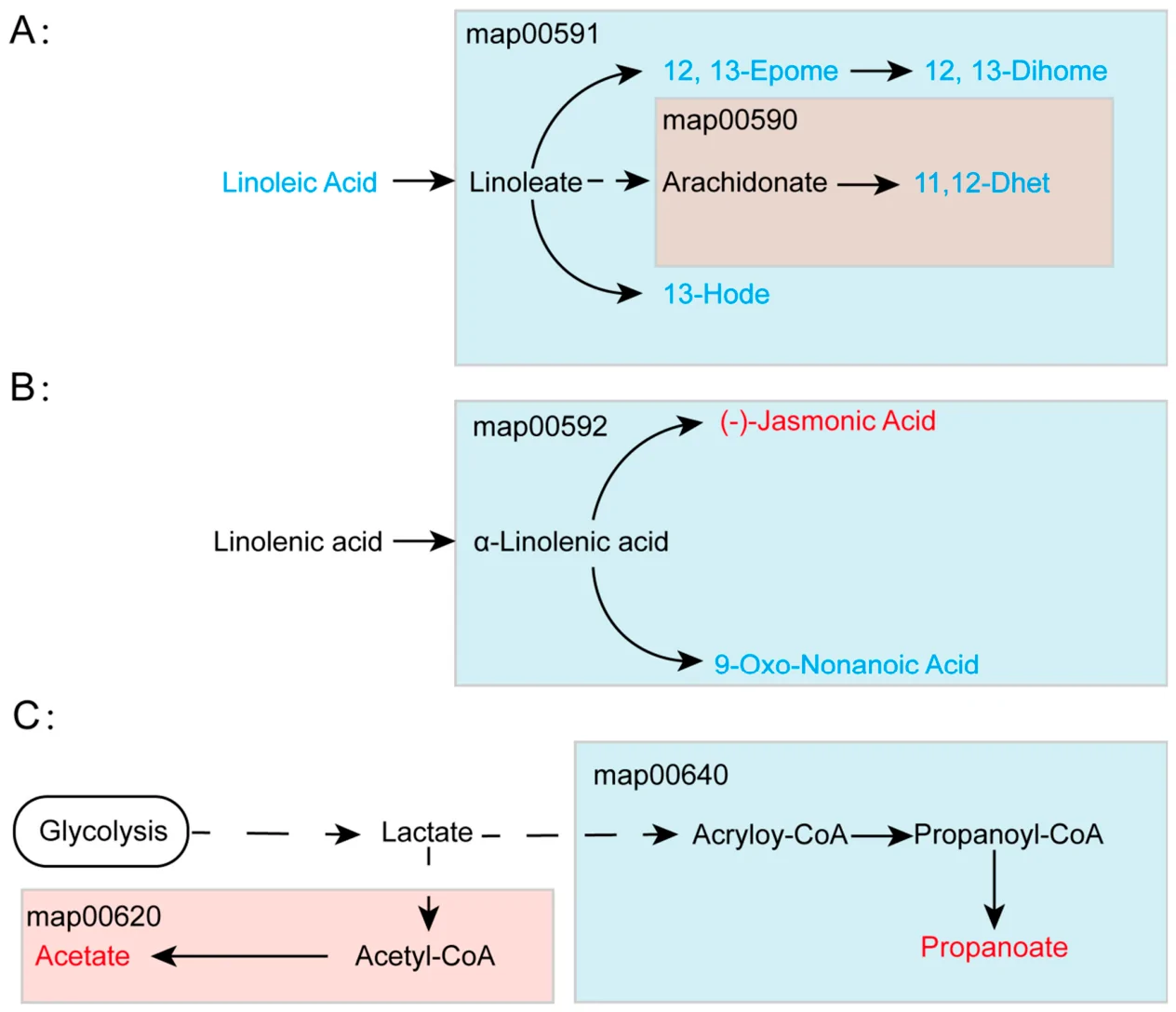
Effects of the GML product on the fecal metabolic pathways of British Shorthair cats. (**A**) Linoleic acid-related metabolic pathways (map00591: Linoleic acid metabolism; map00590: Arachidonic acid metabolism); (**B**) Linolenic acid-related metabolic pathways (map00592: alpha-Linolenic acid metabolism); (**C**) Propionate-related metabolic pathways (map00640: Propanoate metabolism; map00620: Pyruvate metabolism). Red indicates significantly increased levels in the experimental group compared with the control group, whereas blue indicates significantly decreased levels.

As shown in Figure 3A, Linoleic Acid is converted into 12,13-Epome, 12,13-Dihome, and 13-Hode via linoleate, and it enters the arachidonic acid metabolism pathway via arachidonate to generate 11,12-Dhet. Figure 3B illustrates the process by which linolenic acid is converted into (-)-Jasmonic Acid and 9-Oxo-Nonanoic Acid via α-linolenic acid. Furthermore, as depicted in Figure 3C, lactic acid derived from glycolysis is ultimately converted into Propionate, and acetic acid also eventually produces Propionate via Acetyl-CoA.

### 3.11. Correlation Analysis

**Figure 4:**
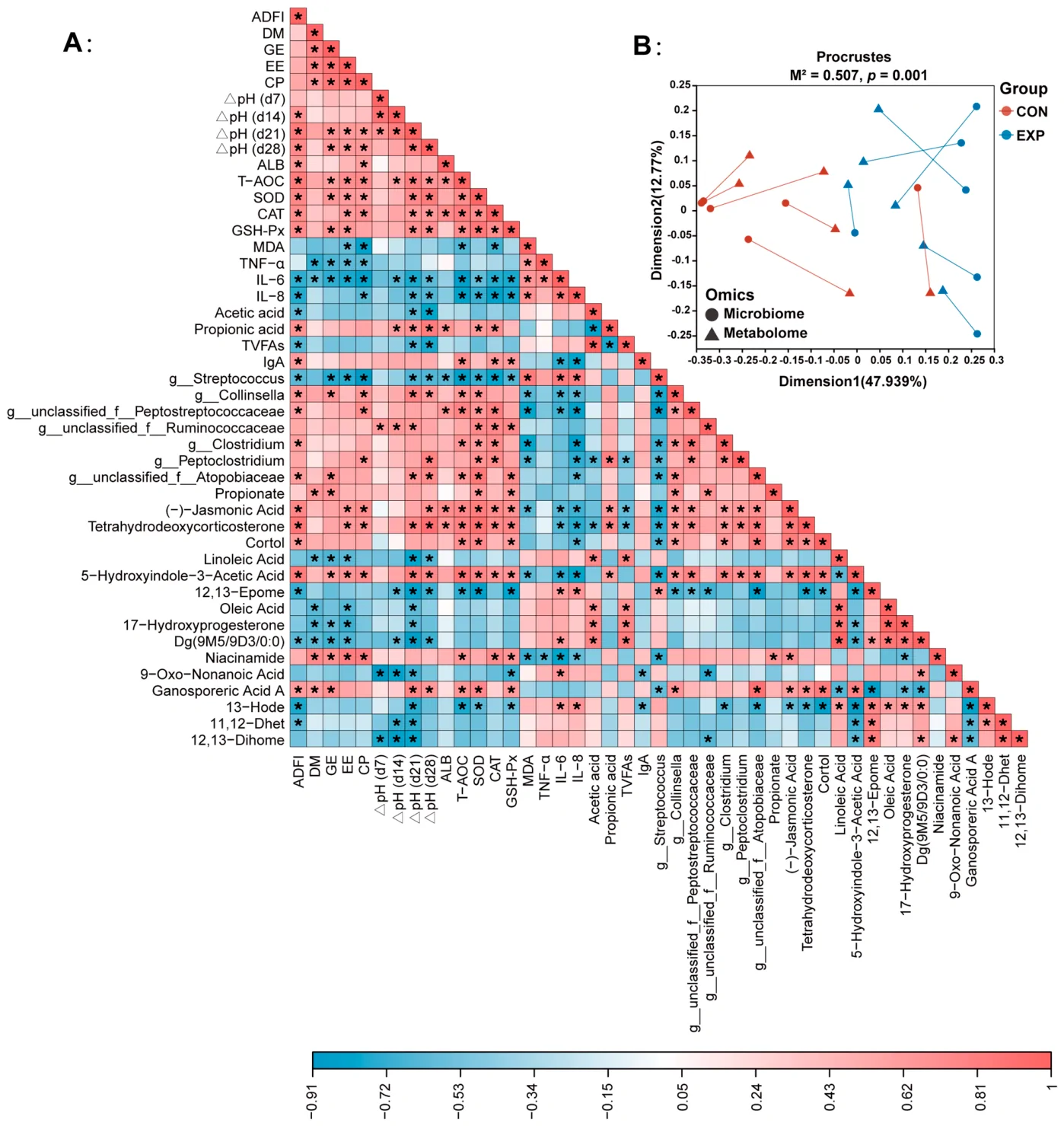
Correlation analysis among the microbiota, metabolites, and phenotypic indices. (**A**) Heatmap of correlations. Red indicates positive correlations, whereas blue indicates negative correlations (* indicates *p* < 0.05). (**B**) Procrustes analysis. Red and blue represent the CON and EXP groups, respectively. The solid circles and triangles indicate the ordination results of the microbiome and metabolome data for the same sample, respectively. The length of the connecting line represents the Procrustes residual, with shorter lines indicating higher concordance between the two datasets (*p* < 0.05 indicates significant concordance). CON: control group; EXP: experimental group; ADFI: average daily feed intake; DM: dry matter; GE: gross energy; EE: ether extract; CP: crude protein; ΔFS: change in fecal score; ΔpH: change in pH; ALB: albumin; T-AOC: total antioxidant capacity; SOD: superoxide dismutase; CAT: catalase; GSH-Px: glutathione peroxidase; MDA: malondialdehyde; TNF-α: tumor necrosis factor-α; IL-6: interleukin-6; IL-8: interleukin-8; TVFAs: total volatile fatty acids; IgA: immunoglobulin A.

The heatmap in Figure 4A illustrates the correlations among the DAMs enriched in the significantly changed metabolic pathways, the fecal microbiota, and phenotypic indices such as performance, apparent nutrient digestibility, and antioxidant function. The abundance of *g__Streptococcus* was significantly and negatively correlated with phenotypic indices including performance, apparent digestibility, pH, antioxidant enzyme activities, and Propionate, whereas it was significantly and positively correlated with MDA, inflammatory cytokines, acetic acid and TVFAs. Furthermore, *g__Streptococcus* was significantly and negatively correlated with several DAMs, including (-)-Jasmonic Acid, Tetrahydrodeoxycorticosterone, Cortol, 5-Hydroxyindole-3-Acetic Acid, Niacinamide, and Ganosporeric Acid A, and was significantly and positively correlated with 12,13-Epome. Additionally, certain correlations were also observed among the other indices. The Procrustes analysis shown in Figure 4B revealed a significant correlation between the fecal microbiota and metabolites (*p* < 0.05).

## 4. Discussion

The intestinal tract of cats is characterized by a short and thick structure, and cats lack salivary amylase and exhibit relatively low activities of pancreatic amylase and intestinal disaccharidases. These characteristics are the physiological manifestations of their obligate carnivorous nature [15]. However, constrained by the manufacturing process of extruded diets, carbohydrates such as starch—for which cats have a limited digestive capacity—are frequently incorporated into the formulations. Because of this limited capacity, prolonged and excessive intake results in undigested carbohydrates entering the hindgut, thereby triggering metabolic disorders and gut microbiota dysbiosis. Similar phenomena have been well-documented in other animals. For instance, a study by You et al. [16] on beef cattle demonstrated that high-grain diets can induce abnormal ruminal fermentation, leading to the excessive production of VFAs, which in turn decreases ruminal pH and causes dysbiosis of the ruminal microbiota. Taken together, previous evidence and the findings of the present study suggest that long-term feeding of high-grain extruded diets may contribute to gut microbiota dysbiosis in cats.

To explore whether the GML product could mitigate this potential negative effect, the present study incorporated it into the extruded cat diet. The results indicated that the body condition scores of cats in the experimental group increased, albeit not significantly; however, the FS and ADFI were significantly improved. This suggests that the GML product may have contributed to the increased feed intake by modulating gut microbial metabolism and intestinal function. Zhao et al. [17] demonstrated that the addition of GML to the diet of mice fed a high-fat diet could regulate the gut microbiota by significantly increasing the abundance of *Bifidobacterium* and decreasing that of *Desulfovibrio*. Furthermore, Kongkeaw et al. [18] reported that feeding a medium-chain triglyceride emulsion rich in GML significantly reduced the diarrhea and mortality rates in weaned piglets, while significantly increasing the ADG. Similarly, Liu et al. [19] found that dietary GML supplementation significantly increased the abundances of *Lachnospiraceae* and *Bifidobacteriaceae* in the cecum of broilers, thereby enhancing the intestinal metabolic capacity for carbohydrates, amino acids, and lipids, which consequently led to significant increases in the ADFI and body weight. These findings also suggest that, in the present study, the supplementation of the GML product may have improved the structure of the feline gut microbiota and enhanced intestinal metabolic capacity, allowing fermentation products to be further degraded for energy supply, which also consequently improved the fecal consistency. Additionally, as a derivative of medium-chain fatty acids, GML can be effectively and rapidly hydrolyzed by gastric and pancreatic lipases, and subsequently rapidly absorbed and utilized by the small intestine for energy production [20]. The synergistic effects of the optimized intestinal environment and improved energy supply may collectively have contributed to the increased feed intake observed in the EXP group.

Apparent nutrient digestibility is a crucial indicator reflecting the capacity of an organism to digest and absorb dietary nutrients. The results of the present study demonstrated that the addition of the GML product to the extruded diet significantly increased the apparent digestibility of dry matter, energy, crude fat, and crude protein in British Shorthair cats. This improvement may be attributed to an amelioration of the feline intestinal structure. Previous studies on other animal species have demonstrated similar effects; for instance, Lin et al. [21] reported that supplementing juvenile pompano (*Trachinotus ovatus*) with GML for 56 days significantly increased the activities of intestinal amylase, lipase, and protease, as well as the intestinal villus length, villus width, and mucosal thickness. Similarly, Wang et al. [11] observed that dietary GML supplementation significantly increased the jejunal villus height and the ratio of villus height to crypt depth in laying hens. Constrained by the welfare and ethical requirements of companion animal trials, the present study did not examine the intestinal morphology of the cats. Therefore, although the observed changes in fecal VFAs and microbiota suggest alterations in the intestinal environment and function, potential effects on intestinal morphology require direct verification. The current findings indicated that the GML product optimized the composition of fermentation products by improving the structure of the microbiota. Specifically, the addition of the GML product reshaped the microbiota, which consequently significantly altered the contents and proportions of fecal VFAs in the cats, manifesting as a significant decrease in the acetic acid content and a significant increase in the Propionate content. Furthermore, from the perspective of energy metabolism, GML, as a derivative of medium-chain fatty acids, is readily hydrolyzed and absorbed [22]. It can exert a synergistic effect with the optimized beneficial fermentation products to provide a more efficient and ample energy substrate for intestinal epithelial cells. This improvement in energy supply facilitates the proliferation and repair of intestinal epithelial cells, thereby expanding the absorptive surface area of the intestine, which ultimately manifests as a significant enhancement in overall digestive capacity. Moreover, this improvement in digestive and absorptive capacity was corroborated by the serum biochemical profile. In the present study, the serum ALB concentration of cats in the experimental group significantly increased within the normal range. Serum ALB, the most abundant plasma protein, reflects the rate of protein digestion and absorption in the body [23]. This corresponds to the significantly increased apparent digestibility of crude protein, indicating that more dietary proteins were effectively degraded, absorbed, and converted into functional proteins of the organism. Additionally, the serum TC and TG levels showed a decreasing trend within the normal range, suggesting that more lipids were catabolized and transported to tissues for absorption and utilization [24], which indirectly reflects an increased rate of fat digestion and absorption in the cats.

Inflammation and oxidative stress are closely interconnected pathophysiological processes. In clinical practice, solely relying on antioxidant therapy may fail to completely reverse the systemic damage induced by oxidative stress; thus, it is usually necessary to combine anti-inflammatory and immunomodulatory interventions to exert synergistic effects [25]. Previous studies have demonstrated that GML exhibits robust anti-inflammatory and antioxidant properties across multiple species. For instance, Kong et al. [9] demonstrated that injecting GML into a chicken embryo model could activate the systemic immune and anti-inflammatory pathways by increasing antioxidant enzyme activities and decreasing the MDA content, thereby reducing the secretion of lipopolysaccharide-induced intestinal inflammatory cytokines. Similarly, Li et al. [26] observed comparable outcomes when supplementing the diet of groupers with GML. Consistent with the findings in the aforementioned species, in the present study, the addition of the GML product to the diet significantly reduced the systemic inflammation levels of the cats, while significantly enhancing their immune and antioxidant capacities. As a derivative of medium-chain fatty acids, GML can freely and rapidly enter the mitochondria for oxidation independent of the carnitine transport system. This highly efficient metabolic mode not only rapidly replenishes cellular energy but also alleviates the load on the mitochondrial electron transport chain, thereby reducing the generation of reactive oxygen species and the subsequent inflammatory responses they induce in the organism [27]. In the serum biochemical profile of the present study, the WBC of cats in the experimental group decreased within the normal range. Although this decrease did not reach statistical significance, it underscores, to a certain extent, the potential of the GML product to mitigate systemic inflammation.

The feline fecal microbiota is closely related to VFA metabolism, immune regulation, and inflammatory responses, serving as a crucial indicator of intestinal physiological structure and function [28,29]. The results of the present study indicated that dietary supplementation with the GML product significantly altered the composition and both the alpha and beta diversities of the feline fecal microbiota. Combined with the taxonomic composition analysis data, it can be observed that the increased microbial population richness in the experimental group was primarily due to the GML-mediated inhibition of the abnormal enrichment of *Streptococcus*. Previous studies have shown that the relative abundance of *Streptococcus* in the intestinal tract of healthy cats is typically maintained at a physiological level of 2% to 20% [28,30,31]. However, in the control group of the present study, which was subjected to long-term feeding of the extruded diet, the abundance of *Streptococcus* reached as high as 65.62%. As obligate carnivores, cats lack salivary amylase and exhibit low activities of pancreatic amylase and intestinal disaccharidases [5,6]; consequently, the long-term feeding of extruded diets results in a large amount of undigested starch entering the hindgut. Although there are currently few studies on starch fermentation by *Streptococcus* in the feline gut, in vitro experiments and other animal models have confirmed that *Streptococcus* possesses the potential to degrade starch [32]. Furthermore, Murray et al. [33] reported that a high-starch diet could induce a significant increase in intestinal *Streptococcus* in horses. Regarding the significantly higher fecal acetic acid concentration in the CON group than in the EXP group, we speculate that this is associated with the extensive fermentation of hindgut starch by *Streptococcus*. This process generates metabolites such as lactic acid, acetic acid, and Propionate [34,35]. However, combined with the metabolomics data of the present study, lactic acid was not significantly enriched in the feces of the control group. This may be because the abnormal fermentation of starch led to a localized drop in intestinal pH, which subsequently inhibited the activities of enzymes related to the synthesis of lactic acid and Propionate [36,37,38], ultimately shifting the metabolic flux of pyruvate generated from glycolysis toward the acetic acid production pathway. Conversely, the GML group improved the structure of the gut microbiota, and the increase in fecal pH created favorable conditions for the colonization of microbiota producing VFAs such as acetic acid, Propionate, and butyric acid, leading to the significant enrichment of various microbial taxa, including *Collinsella*, *Clostridium*, and *Ruminococcaceae*. These microbial taxa can further convert excess acetic acid in the body into secondary metabolites such as Propionate and butyric acid [39,40,41,42]. Additionally, the multi-omics correlation analysis revealed that these alterations in microbial composition and VFAs following GML supplementation could promote the antioxidant, anti-inflammatory, and immune functions of the organism, thereby increasing apparent nutrient digestibility and ultimately improving the feed intake of the cats.

To further elucidate the in vivo mechanisms underlying the efficacy of the GML product, untargeted metabolomics was performed on the feline feces in the present study. The results revealed that, in the GML-supplemented group, several metabolites associated with the lipoxygenase-mediated oxidation of Linoleic Acid were significantly decreased in feces. This decrease may be attributed to a reduction in the Linoleic Acid substrate, suggesting that GML might promote the direct energy supply of Linoleic Acid via β-oxidation in the small intestine. Unlike the small intestinal regulatory model for Linoleic Acid, the metabolic regulation of α-linolenic acid by GML may primarily occur in the hindgut. The results demonstrated that the pro-inflammatory oxidation product 9-Oxo-Nonanoic Acid [43] was significantly decreased in the feces of the experimental group, whereas the anti-inflammatory product (-)-Jasmonic Acid [44] was significantly increased. These changes suggest a potential shift in α-linolenic acid metabolism from the pro-inflammatory branch toward the anti-inflammatory branch. Early studies have indicated that the feline liver inherently lacks Δ6 and Δ5 desaturases, resulting in a limited capacity to further synthesize arachidonic acid from Linoleic Acid, as well as to synthesize eicosapentaenoic acid and docosahexaenoic acid from α-linolenic acid [15,45]. However, recent studies have also demonstrated that cats may exhibit Δ5-desaturase activity under specific dietary conditions to further process high dietary levels of linolenic acid and Linoleic Acid [46]. In the present study, the finding that cats in the GML-supplemented group improved their intestinal environment by altering the metabolic pathways and products of linolenic acid and Linoleic Acid corroborates these recent studies on polyunsaturated fatty acid metabolism. Furthermore, the significant increase in Propionate detected by untargeted metabolomics was consistent with the fecal VFA results. In the relevant metabolic pathways, lactate can contribute to acetate production through intermediates such as pyruvate and can also enter the Propanoate metabolism pathway for Propionate synthesis [47,48]. Lactate can be rapidly utilized by intestinal microorganisms [49]; therefore, the absence of a significant change in fecal lactate does not necessarily indicate unchanged lactate metabolism. In the present study, the significant decrease in acetate accompanied by an increase in Propionate may suggest that GML altered the metabolic allocation of lactate and related carbon substrates, favoring Propionate production. However, the underlying mechanism requires further investigation.

Several limitations of the present study should be acknowledged. First, the relatively small sample size may limit the statistical power and generalizability of the findings. Second, only one GML supplementation level and a 28-day experimental period were evaluated; therefore, the dose-dependent and long-term effects of GML require further investigation. Notably, the final body weight showed an increasing trend in the GML group, and whether this effect would become significant with a longer supplementation period remains to be determined. Third, due to the welfare and ethical constraints of companion animal experiments, intestinal morphology and other direct indicators of intestinal function were not examined. Further in vitro cultivation and transplantation of candidate bacteria will also be needed to clarify their roles in the effects of GML in cats.

## 5. Conclusions

In summary, the present study demonstrated the beneficial effects of the GML product on cats subjected to long-term feeding of extruded diets and provided evidence for the potential biological processes associated with these effects. Specifically, the GML product improved the intestinal environment, which was associated with increased intestinal pH, inhibition of the abnormal enrichment of *Streptococcus*, and changes in acetic acid and Propionate metabolism. These intestinal changes were accompanied by enhanced antioxidant, anti-inflammatory, and immune functions, together with alterations in α-linolenic acid and Linoleic Acid metabolism. Collectively, these changes were associated with significant increases in feed intake and the apparent digestibility of dry matter, energy, crude fat, and crude protein. These findings suggest a potential link among the intestinal environment, gut microbiota, metabolite profiles, and the beneficial physiological effects of the GML product, although the causal relationships and underlying mechanisms require further investigation. The findings of this study provide a theoretical basis for the application of GML products in companion animals and offer insights into the utilization of extruded diets in pet nutrition.

## Figures and Tables

**Table 1:** Composition and nutrient levels of the basal diet (%, dry matter basis).

Items	Content
**Ingredients**	
Chicken powder	28.00
Corn	29.00
Soybean meal	14.00
Rice	23.00
Beet pulp	3.00
Choline chloride	0.50
Monocalcium phosphate	0.80
Potassium chloride	0.30
Taurine	0.20
Calcium propionate	0.20
Premix ^1^	1.00
Total	100.00
**Nutrient levels ^2^**	
CP	29.50
EE	12.65
Ca	1.48
TP	0.89

CP: crude protein; EE: ether extract; Ca: calcium; TP: total phosphorus. ^1^ Provided per kilogram of diet: Vitamin A, 20,000 IU; Vitamin D_3_, 1300 IU; Vitamin E, 120 IU; Vitamin K_3_, 2 mg; Vitamin B_1_, 10 mg; Vitamin B_2_, 10 mg; Vitamin B_6_, 8 mg; Vitamin B_12_, 0.10 mg; Calcium pantothenate, 200 mg; Nicotinic acid, 120 mg; D-pantothenic acid, 34 mg; Folic acid, 2 mg; Biotin, 0.25 mg; Iron, 80 mg; Zinc, 100 mg; Manganese, 28 mg; Copper, 18 mg; Iodine, 3 mg; Selenium, 0.30 mg. ^2^ The concentrations of CP, EE, Ca, and TP were determined by laboratory analysis of the complete basal diet.

**Table 2:** Effects of the GML product on the growth performance of British Shorthair cats (g/d).

Item	CON	EXP	*p*-Value
ADFI	65.70 ± 0.79	70.04 ± 1.12	0.010
ADG	7.32 ± 0.99	10.54 ± 1.50	0.104

CON: control group; EXP: experimental group; ADFI: average daily feed intake; ADG: average daily gain.

**Table 3:** Initial and final body weights and baseline body condition and fecal scores of cats in the two groups.

Item	Day	CON	EXP	*p*-Value
IBW (g)	1	3000.00 ± 89.44	3163.33 ± 87.05	0.220
FBW (g)	28	3205.00 ± 69.51	3458.00 ± 100.21	0.065
BCS	1	4.56 ± 0.41	4.47 ± 0.24	0.864
MCS	1	3.08 ± 0.10	3.12 ± 0.27	0.902
BFI	1	28.97 ± 0.77	29.63 ± 2.02	0.768
FS	1	3.50 ± 0.25	3.25 ± 0.13	0.395
pH	1	5.75 ± 0.05	5.82 ± 0.09	0.521

CON: control group; EXP: experimental group; IBW: initial body weight; FBW: final body weight; BCS: body condition score; MCS: muscle condition score; BFI: body fat index; FS: fecal score.

**Table 4:** Effects of the GML product on the body and fecal parameters of British Shorthair cats.

Item		ΔBCS	ΔMCS	ΔBFI	ΔFS	ΔpH	ΔBW
GML(−)	d7	0.06 ± 0.26	0.00 ± 0.19	0.36 ± 0.36	0.04 ± 0.03	0.02 ± 0.02	98.33 ± 42.69
	d14	0.17 ± 0.19	−0.01 ± 0.06	0.73 ± 0.54	0.06 ± 0.22	0.07 ± 0.01	195.00 ± 19.79
	d21	0.11 ± 0.14	0.08 ± 0.17	1.14 ± 0.78	0.06 ± 0.24	0.05 ± 0.02	215.00 ± 19.45
	d28	0.12 ± 0.07	0.13 ± 0.09	1.22 ± 0.59	0.10 ± 0.05	0.04 ± 0.01	205.00 ± 27.66
GML(+)	d7	0.10 ± 0.13	0.07 ± 0.21	0.50 ± 0.44	0.15 ± 0.07	0.08 ± 0.02 ^d^	86.67 ± 26.42
	d14	0.19 ± 0.18	0.10 ± 0.21	0.97 ± 0.95	0.21 ± 0.14	0.16 ± 0.02 ^c^	168.33 ± 34.97
	d21	0.14 ± 0.15	0.38 ± 0.24	2.61 ± 0.94	0.54 ± 0.27	0.31 ± 0.02 ^b^	228.33 ± 54.37
	d28	0.28 ± 0.07	0.32 ± 0.12	2.92 ± 1.00	0.79 ± 0.18	0.38 ± 0.04 ^a^	295.00 ± 42.09
Main effect							
GML	GML(−)	0.11	0.05	0.86	0.06 ^B^	0.04 ^B^	178.33
	GML(+)	0.18	0.22	1.75	0.42 ^A^	0.23 ^A^	194.58
Day	d7	0.08	0.04	0.43 ^B^	0.09	0.05 ^C^	92.5 ^C^
	d14	0.18	0.04	0.85 ^AB^	0.14	0.11 ^B^	181.67 ^B^
	d21	0.13	0.23	1.88 ^A^	0.30	0.18 ^A^	221.67 ^AB^
	d28	0.20	0.23	2.07 ^A^	0.44	0.21 ^A^	250.00 ^A^
*p*-value							
GML		0.665	0.348	0.215	0.0499	<0.001	0.690
Day		0.826	0.331	0.048	0.100	<0.001	<0.001
GML × Day		0.957	0.854	0.517	0.197	<0.001	0.113

^a,b,c,d^ Within a column, means with different superscript letters were significantly different (*p* < 0.05) among different time points within the same treatment group. ^A,B,C^ Within a column, means with different superscript letters were significantly different (*p* < 0.05) among different levels in the main-effect analysis. GML: glycerol monolaurate; GML(−): control group; GML(+): experimental group; ΔBCS: change in body condition score; ΔMCS: change in muscle condition score; ΔBFI: change in body fat index; ΔFS: change in fecal score; ΔpH: change in pH.

**Table 5:** Effects of the GML product on apparent nutrient digestibility of British Shorthair cats.

Item	CON	EXP	*p*-Value
DM	75.20 ± 1.03	78.89 ± 1.29	0.0495
GE	83.05 ± 0.78	85.41 ± 0.82	0.014
EE	90.84 ± 0.99	94.95 ± 0.47	0.004
CP	76.56 ± 1.27	81.53 ± 0.69	0.006

CON: control group; EXP: experimental group; DM: dry matter; CP: crude protein; EE: ether extract; GE: gross energy.

**Table 6:** Effects of the GML product on the hematological indices of British Shorthair cats.

Item	Normal Range	CON	EXP	*p*-Value
WBC (×10^9^/L)	3.50–19.50	16.25 ± 0.14	14.50 ± 0.92	0.091
Lymph# (×10^9^/L)	0.90–7.00	4.47 ± 0.58	4.25 ± 0.48	0.778
Lymph% (%)	7.00–50.00	31.65 ± 5.07	31.18 ± 4.45	0.946
Mon# (×10^9^/L)	0.04–0.47	0.43 ± 0.02	0.40 ± 0.02	0.207
Mon% (%)	0.80–5.50	3.06 ± 0.21	3.46 ± 0.37	0.364
Gran# (×10^9^/L)	1.90–10.80	10.05 ± 0.47	9.27 ± 1.72	0.670
Gran% (%)	29.00–80.00	62.23 ± 5.43	60.73 ± 5.33	0.848
Eos% (%)	0.40–11.00	6.32 ± 0.98	6.33 ± 0.89	0.990
MCV (fL)	36.00–48.00	44.65 ± 1.15	43.93 ± 1.03	0.675
MCH (pg)	12.00–18.50	14.95 ± 0.40	14.45 ± 0.42	0.408
MCHC (g/L)	300.00–400.00	333.17 ± 1.82	334.50 ± 0.50	0.495
RDW (%)	16.00–30.00	16.25 ± 0.25	16.22 ± 0.21	0.921
RBC (×10^12^/L)	5.00–11.50	8.92 ± 0.67	8.84 ± 0.32	0.916
HGB (g/L)	85.00–175.00	127.33 ± 5.26	128.17 ± 6.39	0.922
HCT (%)	26.00–47.00	39.55 ± 2.08	38.83 ± 1.78	0.799
PLT (×10^9^/L)	100.00–500.00	197.83 ± 1.92	198.33 ± 2.56	0.879
MPV (fL)	7.40–14.00	9.75 ± 0.44	9.65 ± 0.17	0.836
PDW (%)	6.50–21.00	15.43 ± 0.53	15.13 ± 0.10	0.587
PCT (%)	0.02–0.80	0.21 ± 0.01	0.20 ± 0.01	0.786

CON: control group; EXP: experimental group; WBC: white blood cell count; Lymph#: lymphocyte count; Lymph%: lymphocyte percentage; Mon#: monocyte count; Mon%: monocyte percentage; Gran#: granulocyte count; Gran%: granulocyte percentage; Eos%: eosinophil percentage; MCV: mean corpuscular volume; MCH: mean corpuscular hemoglobin; MCHC: mean corpuscular hemoglobin concentration; RDW: red blood cell distribution width; RBC: red blood cell count; HGB: hemoglobin concentration; HCT: hematocrit; PLT: platelet count; MPV: mean platelet volume; PDW: platelet distribution width; PCT: plateletcrit.

**Table 7:** Effects of the GML product on the serum biochemical indices of British Shorthair cats.

Item	Normal Range	CON	EXP	*p*-Value
BUN (mg/dL)	16.00–36.00	20.91 ± 0.66	21.71 ± 1.16	0.561
TP (g/L)	54.00–89.00	78.55 ± 1.36	80.21 ± 1.93	0.498
ALB (g/L)	25.00–45.00	28.54 ± 0.68	31.62 ± 1.13	0.041
GLB (g/L)	15.00–57.00	50.01 ± 1.45	48.60 ± 1.87	0.563
TC (mmol/L)	1.68–5.81	2.84 ± 0.26	2.10 ± 0.24	0.061
TG (mmol/L)	0.10–0.90	0.46 ± 0.04	0.36 ± 0.03	0.068
HDL (mmol/L)	0.80–2.00	1.81 ± 0.16	1.85 ± 0.22	0.882
ALP (U/L)	10.00–90.00	28.45 ± 4.59	29.31 ± 4.17	0.892
AST (U/L)	10.00–40.00	36.97 ± 2.46	36.76 ± 2.44	0.954
ALT (U/L)	8.20–123.00	49.69 ± 0.97	49.69 ± 1.25	0.996
TBIL (umol/L)	2.00–15.00	2.86 ± 0.20	2.73 ± 0.16	0.614
γ-GT (U/L)	0.00–2.00	1.49 ± 0.40	1.30 ± 0.12	0.659

CON: control group; EXP: experimental group; BUN: blood urea nitrogen; TP: total protein; ALB: albumin; GLB: globulin; TC: total cholesterol; TG: triglycerides; HDL: high-density lipoprotein; ALP: alkaline phosphatase; AST: aspartate aminotransferase; ALT: alanine aminotransferase; TBIL: total bilirubin; γ-GT: gamma-glutamyl transferase.

**Table 8:** Effects of the GML product on the serum antioxidant function of British Shorthair cats.

Item	CON	EXP	*p*-Value
T-AOC (U/mL)	8.82 ± 0.04	10.79 ± 0.04	<0.001
SOD (U/mL)	63.58 ± 1.27	78.33 ± 1.61	<0.001
CAT (U/mL)	34.90 ± 1.67	48.54 ± 1.14	<0.001
GSH-Px (U/mL)	130.78 ± 3.53	170.06 ± 2.43	<0.001
MDA (nmol/mL)	4.17 ± 0.45	2.83 ± 0.29	0.030

CON: control group; EXP: experimental group; T-AOC: total antioxidant capacity; SOD: superoxide dismutase; GSH-Px: glutathione peroxidase; CAT: catalase; MDA: malondialdehyde.

**Table 9:** Effects of the GML product on the serum immune function of British Shorthair cats.

Item	CON	EXP	*p*-Value
IgA (g/L)	1.30 ± 0.04	1.48 ± 0.05	0.024
IgG (g/L)	8.24 ± 0.57	8.73 ± 0.46	0.521
IgM (g/L)	0.90 ± 0.06	0.96 ± 0.05	0.494
TNF-α (pg/mL)	58.06 ± 4.70	44.33 ± 3.46	0.040
IL-6 (pg/mL)	174.31 ± 6.12	116.84 ± 4.79	<0.001
IL-8 (pg/mL)	107.17 ± 2.48	78.55 ± 3.56	<0.001

CON: control group; EXP: experimental group; IgA: immunoglobulin A; IgG: immunoglobulin G; IgM: immunoglobulin M; TNF-α: tumor necrosis factor-α; IL-6: interleukin-6; IL-8: interleukin-8.

**Table 10:** Effects of the GML product on the fecal volatile fatty acids of British Shorthair cats (μg/g).

Item	CON	EXP	*p*-Value
Acetic acid	5273.57 ± 136.14	4540.06 ± 206.66	0.014
Propionic acid	284.73 ± 13.92	373.87 ± 26.02	0.014
Isobutyric acid	12.42 ± 2.02	15.99 ± 2.17	0.257
Butyric acid	275.64 ± 34.32	310.22 ± 31.92	0.478
Isovaleric acid	23.74 ± 6.36	28.65 ± 8.08	0.644
Valeric acid	110.78 ± 6.11	123.27 ± 10.76	0.337
TVFAs	5980.88 ± 151.83	5391.06 ± 199.38	0.040

CON: control group; EXP: experimental group; TVFAs: total volatile fatty acids.

## Data Availability

The 16S rRNA sequencing data have been deposited in the NCBI Sequence Read Archive under BioProject accession number PRJNA1481144.
